# Modeling genetic imprinting effects of DNA sequences with multilocus polymorphism data

**DOI:** 10.1186/1748-7188-4-11

**Published:** 2009-08-11

**Authors:** Sheron Wen, Chenguang Wang, Arthur Berg, Yao Li, Myron M Chang, Roger B Fillingim, Margaret R Wallace, Roland Staud, Lee Kaplan, Rongling Wu

**Affiliations:** 1 Department of Statistics, University of Florida, Gainesville, Florida 32611, USA; 2 Department of Epidemiology and Health Policy Research, University of Florida, Gainesville, Florida 32611, USA; 3 Department of Community Dentistry and Behavioral Science, University of Florida, Gainesville, Florida 32611, USA; 4 Department of Molecular Genetics and Microbiology, University of Florida, Gainesville, Florida 32611, USA; 5 Department of Public Health Sciences, Pennsylvania State College of Medicine, Hershey, Pennsylvania 17033, USA; 6 Department of Statistics, Pennsylvania State University, University Park, Pennsylvania 16802, USA

## Abstract

Single nucleotide polymorphisms (SNPs) represent the most widespread type of DNA sequence variation in the human genome and they have recently emerged as valuable genetic markers for revealing the genetic architecture of complex traits in terms of nucleotide combination and sequence. Here, we extend an algorithmic model for the haplotype analysis of SNPs to estimate the effects of genetic imprinting expressed at the DNA sequence level. The model provides a general procedure for identifying the number and types of optimal DNA sequence variants that are expressed differently due to their parental origin. The model is used to analyze a genetic data set collected from a pain genetics project. We find that DNA haplotype GAC from three SNPs, OPRKG36T (with two alleles G and T), OPRKA843G (with alleles A and G), and OPRKC846T (with alleles C and T), at the kappa-opioid receptor, triggers a significant effect on pain sensitivity, but with expression significantly depending on the parent from which it is inherited (*p *= 0.008). With a tremendous advance in SNP identification and automated screening, the model founded on haplotype discovery and statistical inference may provide a useful tool for genetic analysis of any quantitative trait with complex inheritance.

## Background

In diploid organisms, there are two copies at every autosomal gene, one inherited from the maternal parent and the other from the paternal parent. Both copies are expressed to affect a trait for a majority of these genes. Yet, there is also a small subset of genes for which one copy from a particular parent is turned off. These genes, whose expression depends on the parent of origin due to the epigenetic or imprinted mark of one copy in either the egg or the sperm, have been thought to play an important role in complex diseases and traits, although imprinted expression can also vary between tissues, developmental stages, and species [1]. Anomalies derived from imprinted genes are often manifested as developmental and neurological disorders during early development and as cancer later in life [2-5].

The mechanisms for genetic imprinting are still incompletely known, but they involve epigenetic modifications that are erased and then reset during the formation of eggs and sperm. Recent research shows that the parent-of-origin dependent expression of imprinted genes is related with environmental interactions with the genome [5]. The phenomenon of genomic imprinting is explained from an evolutionary perspective [6]. Genomic imprinting evolves in mammals with the advent of live birth through a parental battle between the sexes to control the maternal expenditure of resources to the offspring [7].

Paternally expressed imprinted genes tend to promote offspring growth by extracting nutrients from the mother during pregnancy while it is suppressed by those genes that are maternally expressed. This genetic battle between the paternal and maternal parents appears to continue even after birth [8,9].

The genetic mechanisms for imprinting can be made clear if the genomic distribution of imprinted genes and their actions and interactions are studied. Genetic mapping with molecular markers and linkage maps has been used to map quantitative trait loci (QTLs) that show parent-of-origin effects [10-12]. Using an outbred strategy appropriate for plants and animals, significant imprinting QTLs were detected for body composition and body weight in pigs [13,14], chickens [15] and sheep [16]. Cui et al. [12] proposed an F_2_-based strategy to map imprinting QTLs by capitalizing on the difference in the recombination fraction between different sexes. More explorations on the development of imprinting models are given in Cui and others [12,17]. Liu et al. [18] developed a random-effect model for estimating the parent-dependent genetic variance of complex traits at imprinting QTLs.

We will propose a statistical model for estimating the imprinting effects of DNA sequence variants that encode a complex trait. This model uses widely available single nucleotide polymorphisms (SNPs) that reside within a coding sequence of the human genome. The central idea of this model is to separate maternally- and paternally-derived haplotypes (i.e., a linear combination of alleles at different SNPs on a single chromosome) from observed genotypes. By specifying one risk haplotype, i.e., one that operates differently from the rest of haplotypes (called non-risk haplotypes), Liu et al. [19] proposed a statistical method for detecting risk haplotypes for a complex trait with a random sample drawn from a natural population. Liu et al.'s approach can be used to characterize DNA sequence variants that encode the phenotypic value of a trait. Wu et al. [20] constructed a general multiallelic model in which any number of risk haplotypes can be assumed. The best number and combination of risk haplotypes can be estimated by using the likelihoods and AIC or BIC values. We will derive a computational algorithm for estimating the imprinting effects of SNP-constructed haplotypes with multilocus genetic data based on these previous workings. The new algorithmic model provides a general framework for hypothesis tests on the pattern of genetic imprinting expressed by haplotypes. A real example from a pain genetic study is used to demonstrate the application of the model.

## Results

The new imprinting model was used to detect the difference of gene expression between the maternal and paternal parents at the haplotype level. Genetic and phenotypic data were from a pain genetics project in which 237 subjects (including 143 men and 94 women) from five different races were sampled. All these subjects were genotyped for three SNPs, OPRKG36T (rs1051160), with two alleles G and T, OPRKA843G (rs702764), with alleles A and G, and OPRKC846T (rs16918875), with alleles C and T, at the kappa-opioid receptor [21]. Three traits of pain sensitivity to heat were tested with a procedure described by Fillingim [22], and they are the average pain rating for heat stimuli at 49°C (PreInt49tot), the increase in heat pain threshold following administration of 0.5 mg/kg of pentazocine (an mixed action opioid agonist-antagonist with activity at the kappa receptor) (Hpthpent), and the increase in heat pain tolerance following administration of 0.5 mg/kg of pentazocine (an mixed action opioid agonist-antagonist with activity at the kappa receptor) (Hptopent). PreInt49tot is a baseline pain measure before any drug administration. Although the model allows the estimation of any covariate effect, we will remove the effect on the pain traits due to different races because of too few samples for some races. Our final imprinting analysis was based on 237 subjects for PreInt49tot but on 85 subjects for Hpthpent and Hptopent. Sex-specific haplotype frequencies for the three SNPs were estimated for males and females, from which linkage disequilibria were estimated and tested with results given in Table 1. Of the eight haplotypes, haplotype GAC occupies an overwhelming proportion in both sexes (>75%). Some haplotypes, like GAT, TAT, and TGC, are very rare, with population frequencies tending to be zero. Linkage disequilibria between these SNPs are generally strong: those between OPRKA843G and OPRKC846T and between OPRKG36T and OPRKC846T are highly significant (*p *< 0.01), although that between OPRKG36T and OPRKA843G is much less significant. There is a highly significant high-order linkage disequilibrium among these three SNPs. It is interesting to see significant difference in haplotype distribution between the two sexes (Table 1). This sex-specific difference is due to the difference in linkage disequilibria because allele frequencies seem to be mostly sex-invariant (Table 1).

**Table 1:** Sex-specific differences observed in haplotype frequencies and higher-order linkage disequilibria estimates

	Male		Female		Sex-specific	Sex-specific
				
Genetic Parameter	MLE	*p*-value	MLE	*p*-value	LR	*p*-value
**Haplotype Frequency**						
*GAC*	0.780		0.764			
*GAT*	0.000		0.000			
*GGC*	0.124		0.081			
*GGT*	0.011		0.023			
*TAC*	0.049		0.104			
*TAT*	0.000		0.000			
*TGC*	0.008		0.000			
*TGT*	0.030		0.029			
						
**Allele Frequency and Linkage Disequilibrium**						
*G *(OPRKG36T)	0.914		0.868		6.166	0.0130
*A *(OPRKA843G)	0.828		0.868		3.501	0.0613
*A *(OPRKC846T)	0.960		0.949		2.943	0.0862
_12_	0.034	0.0459	0.045	0.1812	5.771	0.0163
_23_	0.026	4.98*e*^-6^	0.022	3.61*e*^-6^	42.281	7.91^*e*-11^
_13_	0.023	8.87*e*^-5^	0.011	0.0089	22.216	2.44*e*^-6^
_123_	-0.021	2.53*e*^-4^	-0.018	0.0055	21.088	4.39*e*^-6^

Estimates and tests of population genetic structure for three SNPs, OPRKG36T (with two alleles G and T), OPRKA843G (with alleles A and G), and OPRKC846T (with alleles C and T), at the kappa-opioid receptor in males and females.

A "biallelic" model assuming that there is only one haplotype is used to estimate haplotype effects at the kappa-opioid receptor on pain traits.

Significant haplotype effects were observed on the three pain sensitivity traits studied. By assuming a risk haplotype from all possible haplotypes, we calculated the resultant likelihoods of haplotype effects, which are given in Table 2.

**Table 2:** Haplotype effects are estimated over three pain sensitivity traits

Trait	GAC	GAT	GGC	GGT	TAC	TAT	TGC	TGT
PreInt49tot	-764.663	-773.196	-771.972	-770.903	-769.645	-773.196	-772.842	-772.677
Hpthpent	-195.107	-199.832	-198.640	-199.460	-193.569	-199.832	-195.345	-199.709
Hptopent	-165.867	-170.494	-170.468	-170.216	-157.053	-170.494	-168.324	-170.414

It can be seen that an optimal risk haplotype is GAC for preint49tot and TAC for Hpthpent and Hptopent. Statistical tests indicate that these risk haplotypes trigger a significant effect on PreInt49tot (*p *= 0.004) and Hpthpent and Hptopent (*p *= 0.025), respectively (Table 3). Risk haplotype GAC displays a significant additive effect (*p *= 0.005) on preint49tot, but there is no dominant effect due to its interaction with non-risk haplotypes. It is interesting to find that risk haplotype GAC contributes to variation in preint49tot in a parent-of-origin manner. The subjects with risk haplotype GAC inherited from the maternal parent will be significantly different in preint49tot than those with the risk haplotype inherited from the paternal parent. No significant imprinting effects are detected on traits Hpthpent and Hptopent, although risk haplotype TAC displays significant additive and dominant effects on these two traits.

**Table 3:** Additive, dominant, imprinting, and overall effects at three SNPs

Trait		Risk Haplotype	*a*	*d*	*i*	Overall
PreInt49tot	Effect	GAC	-13.47	-6.22	19.02	
	*p*-value		0.005	0.237	0.008	0.004
						
Hpthpent	Effect	TAC	3.06	3.08	-0.26	
	*p*-value		0.002	0.003	0.089	0.023
						
Hptopent	Effect	TAC	2.15	2.33	-0.28	
	*p*-value		0.003	0.003	0.621	0.025

Estimates of additive (*a*), dominant (*d*), and imprinting effects (*i*) of haplotypes at SNPs, OPRKG36T (with two alleles G and T), OPRKA843G (with alleles A and G), and OPRKC846T (with alleles C and T), at the kappa-opioid receptor, on pain sensitivity traits.

The statistical properties of the imprinting model are investigated through simulation studies. The first simulation mimics the data structure of the real example (with 237 subjects) above based on its estimates of sex-specific allele frequencies and linkage disequilibria among three SNPs (Table 1) and of the additive, dominant, and imprinting effects arising from different haplotypes (for PreInt49tot, Table 3). The second simulation includes sample size from 237 to 400, 800, and 2000, keeping the other parameters unchanged. Each simulation scheme is repeated for 1000 runs.

Results from simulation studies are summarized as follows:

(1) Population genetic parameters including three-SNP haplotype frequencies, allele frequencies, and linkage disequilibria of different orders can be precisely estimated even when a smaller sample size (237) is used. As expected, the estimation precision can be improved consistently when the sample size increases from 237 to 2000.

(2) Quantitative genetic parameters can also be well estimated, but the better estimation of the dominant and imprinting effects needs a larger sample size (400 or more). With a sample size of 2000, the precision of all parameter estimates are remarkably high, with sampling errors of each estimate being low than 10% of the estimate.

(3) The power to detect imprinting effects reaches 75% for a sample size of 237, but it can increase dramatically when increasing the sample size to 400.

(4) The type I error rate of detecting the imprinting effect, i.e, a probability for a false positive discovery of that effect, is quite low (< 10%) for a small sample size and can be lowered when sample size increases.

The simulation for testing the type I error rate in (4) was based on the same parameters as used in (1)–(3), except that no imprinting effect is assumed. Because we have derived a series of closed forms for the estimation of parameters within the EM framework, parameter estimation is very efficient. For a single simulation run, it will take a few dozen of seconds to obtain all estimates on a PC laptop. Also, estimates do not depend heavily on initial values of parameters, showing that the estimates achieve a global maxima for the likelihood.

## Discussion

Although a traditional view assumes that the maternally and paternally derived alleles of each gene are expressed simultaneously at a similar level, there are many exceptions where alleles are expressed from only one of the two parental chromosomes [1,23]. This so-called genetic imprinting or parent-of-origin effect has been thought to play a pivotal role in regulating the phenotypic variation of a complex trait [24-27]. With the discovery of more imprinting genes involved in trait control through molecular and bioinformatics approaches, we will be in a position to elucidate the genetic architecture of quantitative variation for various organisms including humans. Genetic mapping in controlled crosses has opened up a great opportunity for a genome-wide search of imprinting effects by identifying imprinted quantitative trait loci (iQTLs). This approach has successfully detected iQTLs that are responsible for body mass and diseases [10,11,14,19,28,29]. Cloning of these iQTLs will require high-resolution mapping of genes, which is hardly met for traditional linkage analysis based on the production of recombinants in experimental crosses. Single nucleotide polymorphisms (SNPs) are powerful markers that can explain interindividual differences. Multiple adjacent SNPs are especially useful to associate phenotypic variability with haplotypes [30-34]. The quantitative effect of haplotypes on a complex trait was modeled by Liu et al. [19] and subsequently refined by Wu et al. [20].

In this article, we incorporate genetic imprinting into Wu et al.'s [20] multiallelic model to estimate the number and combination of multiple functional haplotypes that are expressed differently depending on the parental origin of these haplotypes. Because of the modeling of any possible distinct haplotypes, the multiallelic model will have more power for detecting significant haplotypes and their imprinting effects than biallelic models. The imprinting model was shown to work well in a wide range of parameter space for a modest sample size. However, a considerably large sample size is needed if there are multiple risk haplotypes that contribute to trait variation. By analyzing a real example for pain genetics, the new model detects significant haplotypes composed of three SNPs within the kappa-opioid receptor, which may play an important role in affecting pain sensitivity to heat.

Haplotype GAC derived from this gene appears to be imprinted for PreInt49tot, a pain sensitivity trait to heat stimuli at 49°C before drug administration, leading to different levels of pain sensitivity between the patients when they inherit this haplotype from maternal and paternal parents. In this example, no imprinting was detected for Hpthpent and Hptopent, two pain sensitivity traits measured after the patients were administrated with pentazocine. This result should be, however, explained with caution. First, the risk haplotype, TAC, detected for Hpthpent and Hptopent is a rare haplotype in the admixed population studied, although it is quite common in African Americans and Hispanics. The significance of this rare haplotype detected could be a sample size artifact, or it could be indicating a powerful haplotype effect. Second, in a different analysis, no significant genetic association was detected for the same heat pain test at heat stimuli at 52°C (data not shown). Nonetheless, the method provides a powerful tool for detecting possible associations and imprinting effects, which provide a starting point for future work to pursue the positive results with larger sample sizes and family studies. There have not been any previous reports suggesting an imprinting effect at an opioid receptor locus, or related to pain measures.

In practice, although the human genome contains millions of SNPs, it is not possible and also not necessary to model and analyze these SNPs simultaneously. These SNPs are often distributed in different haplotype blocks [35], within each of which a particular (small) number of representative SNPs or htSNPs can uniquely explain most of the haplotype variation. A minimal subset of htSNPs, identified by several computing algorithms, can be implemented into our imprinting model to detect their imprinting effects at the haplotype level. In addition, our model can be extended to model imprinting effects in a network of interactive architecture, including haplotype-haplotype interactions from different genomic regions, haplotype-environment interactions, and haplotype effects regulating pharmacodynamic reactions of drugs. It can be expected that all extensions will require expensive computation, but this computing can be made possible if combinatorial mathematics, graphical models, and machine learning are incorporated into closed forms of parameter estimation.

This imprinting model assumes that if the SNPs constituting haplotypes are tightly linked, haplotype frequencies estimated from the current generation can be used to approximate haplotype frequencies in the parental generation. To relax this assumption, a strategy of sampling a panel of random families from a population is required, in which a known family structure allows the tracing and estimation of maternally- and paternally-derived haplotypes. Such a strategy will help to precisely estimate and test imprinting effects of haplotypes, providing a new gateway for studying the genetic architecture of complex traits.

## Methods

### Imprinting Model

Consider a set of three ordered SNPs, each with two alleles 1 and 0, genotyped from a candidate gene. These three SNPs form eight haplotypes, 111, 110, 101, 100, 011, 010, 001, and 000. A risk haplotype group is defined as a set of haplotypes that are in manner distinct from the other haplotypes in affecting a complex trait. For example, if a risk haplotype group only consists of the haplotype 111, the remaining seven haplotypes form the non-risk haplotype group; this means that the diplotypes composed of 111 will have different genotypic values from those composed of 110, 101, 100, 011, 010, 001, or 000. There may be multiple risk haplotype groups, and we let *R*_*k *_denote any risk haplotype from the *k*^th ^risk haplotype group (*k *= 1,...,*K*; *K *< 8, where *K *is the number of risk haplotype groups) and *R*_0 _denote any of the remaining non-risk haplotypes in the non-risk haplotype group. The combinations between the risk and non-risk haplotypes are called the composite diplotypes, including *R*_*k*_*R*_*k' *_(*k *≤ *k' *= 1,...,*K*), *R*_*k*_*R*_0 _and *R*_0_*R*_0 _(any two non-risk haplotypes). Here we do not distinguish between *R*_*k*_*R*_*k' *_and *R*_*k'*_*R*_*k *_as we do not know parental origin of the haplotypes, however genetic imprinting implies that the same composite diplotype may function differently, depending on the parental origin of its underlying haplotypes. To reflect the parental origin of haplotypes in the composite diplotype, the following notation is used: *R*_*k*_|*R*_*k' *_(*k*, *k' *= 1,....,*K*), *R*_*k*_|*R*_0_, *R*_0_|*R*_*k*_, and *R*_0_|*R*_0_, where the vertical lines are used to separate the haplotypes derived from the maternal parent (left) and paternal parent (right).

According to traditional quantitative genetic principles, the genotypic value of a given composite diplotype is partitioned into different components due to additive and dominance genetic effects. For an imprinting model, an additional component is the imprinting genetic effect due to different contributions of haplotypes from the maternal and paternal parents. Mathematically, the genotypic value of a composite diplotype of known parental origins is expressed as



where *μ *is the overall mean, *a*_*k *_is the additive effect due to the substitution of risk haplotype *k *by the non-risk haplotype, *d*_*kk' *_is the dominance effect due to the interaction between risk haplotypes *k *and *k'*(*d*_*kk' *_= *d*_*k*'*k*_), *d*_*k*0 _is the dominance effect due to the interaction between risk haplotypes *k *and the non-risk haplotype (*d*_*k*0 _= *d*_0*k*_), *i*_*kk' *_is the imprinting effects due to different parental origins of risk haplotypes *k *and *k'*(*i*_*kk' *_= *i*_*k'k*_), and *i*_*k*0 _is the imprinting effect due to different parental origins of risk haplotype *k *and the non-risk haplotype (*i*_*k*0 _= *i*_0*k*_). The sizes and signs of *i*_*kk' *_and *i*_*k*0 _determine the extent and direction of imprinting effects at the haplotype level.

A set of genetic parameters, including the additive, dominance, and imprinting effects, define the genetic architecture of a quantitative trait. By estimating and testing these parameters, the genetic architecture of a trait can well be studied. Specific genetic effects of haplotypes can be estimated from genotypic values of the composite diplotypes with the formulas as follows:



### Estimating Model

#### Genetic Structure

Suppose the three SNPs are genotyped from a natural human population at Hardy-Weinberg equilibrium (HWE). Let  and  denote the frequencies of two alternative alleles *r*_*l *_(*r*_*l *_= 1 or 0) at SNP *l *in the population of sex *s *(*s *= *M *for females and *P *for males). Sex-specific frequencies of eight haplotypes produced by the three SNPs are generally expressed as . For each sex *s*, genotypes consisting of three SNPs are denoted as , totaling 27 distinct genotypes. Let  denote the observation of a three-SNP genotype for sex *s*, which sums over the two sexes to . Some genotypes are consistent with diplotypes, whereas those that are heterozygous at two or more SNPs are not. Each double heterozygote contains two different diplotypes, and the triple heterozygote, i.e., 10/10/10, contains four different diplotypes: 111|000 (with probability  for sex *s*), 110|001 (with probability  for sex *s*), 101|010 (with probability  for sex *s*), and 100|011 (with probability  for sex *s*). Note that slashes are used to separate genotypes at different SNPs and vertical lines are used to separate haplotypes derived from the maternal parent (left) and paternal parent (right). The observed genotypes, the underlying diplotypes, and diplotype frequencies are provided in Additional file 1. From the HWE assumption, diplotype frequencies are simply expressed as the products of the underlying-haplotype frequencies derived from different parents.

For a random sample from a natural population, it is impossible to estimate the frequencies of maternally- and paternally-derived haplotypes. However, parent-specific haplotype frequencies can be approximated by sex-specific haplotype frequencies in the current generation if the SNPs studied are highly linked together. For example, we will argue below that  which measures frequency for the haplotype 100 among females in the current generation can be accurately approximated by the haplotype frequency 100 in the "maternal generation" or previous generation of females. This is proven as follows. Let (*t*) and (*t *+ 1) be the frequencies of a representative three-SNP haplotype *r*_1_*r*_2_*r*_3 _for a monosexual population in generations *t *and *t *+ 1, respectively. The relationship of haplotype frequencies between the two generations is expressed as



where (*t*), (*t*), and (*t*) are the allele frequencies of three SNPs in generation *t*, and *D*_12_(*t*), *D*_13_(*t*), and (*D*_23_(*t*) are the linkage disequilibria between the first and second SNPs, the first and third SNPs, and the second and third SNPs in generation *t*. Under Hardy-Weinberg equilibrium, the allele frequencies remain constant from generation to generation  and the linkage disequilibria decay in proportion with the recombination fractions *r*_*ij *_in that



and



Because the three SNPs are genotyped from the same region of a candidate gene, their recombination fractions should be very small and can be thought to be close to zero. Thus, the frequencies of maternally- and paternally-derived haplotypes can be approximated by the estimates of these haplotype frequencies in the female and male populations of current generation, respectively.

#### Likelihoods

With a random sample from a natural population, in which each genotyped subject is measured for a phenotypic trait of interest, we will develop a model to estimate population genetic parameters, including the eight maternally-derived haplotype frequencies , the eight paternally-derived haplotype frequencies , and the quantitative genetic parameters (**Ω**_*q*_) that include haplotype effects () and the residual variance of the trait (*σ*^2^). The haplotype effects are derived uniquely from genotypic values of composite diplotypes () as provided in equations (2)–(6).

Given sex-specific genotypic observations (**M**_*M *_and **M**_*P*_) and phenotypic data (*y*), a joint log-likelihood is constructed as



Thus, maximizing the likelihood in (1) is equivalent to individually maximizing the three terms on the right hand side of (1).

A polynomial likelihood is constructed for the marker data of a sex (*s*) to estimate  and . For notational convenience, we define a function *h*(***r***) on genotypes  to be



So, for example, *h*(***r***) = 2 if ***r ***is a double heterozygote. The first two log likelihoods on the righthand side of equation (7) are then expressed as



A closed system of the EM algorithm was derived to estimate these haplotype frequencies (see the Text S1). The estimates of sex-specific haplotype frequencies are then used to estimate haplotype effects by constructing a mixture model-based likelihood. The construction of this likelihood requires knowledge of which haplotypes are risk haplotype(s). By assuming that haplotype 111 is only one risk heplotype, we construct the likelihood as



and



The EM algorithm is derived to estimate quantitative genetic parameters with a detailed procedure given in Additional file 2. The estimated genotypic values of composite diplotypes are used to estimate the additive, dominant and imprinting effects of haplotypes using equations (2)–(6).

### Model Selection

For an observed marker (**M**) and phenotypic (*y*) data set, we do not know which are the risk haplotypes nor how many there are. Standard model selection criteria such as the AIC and BIC are used to determine the optimal number and type of risk haplotypes. Among a total of eight haplotypes formed by three SNPs, up to seven ones can be risk haplotypes. The modeling of one to seven risk haplotypes is equivalent to the analysis of the genetic data by biallelic, triallelic, quadrialleli, pentaallelic, hexaallelic, septemallelic and octoallelic models, respectively. The biallelic model dissolves all the haplotypes into two distinct groups, a single risk haplotype group and a non-risk haplotype group. The risk haplotype group may be composed of one (8 choices), two (28 choices), three (56 choices) or four haplotypes (70 choices). The triallelic, quadrialleli, pentaallelic, hexaallelic, septemallelic and octoallelic models contains 28, 56, 170, 56, 24 and 8 cases, respectively. The likelihoods and model selection criteria, AIC or BIC, are then calculated and compared among different models and assumptions. This is summarized in Table 4.

**Table 4:** Number of choices for serveral multiallelic models with likelihood and model selection notation

		Risk Haplotype	Log-likelihood	AIC/BIC
			
Model	No.	Choice		
Biallelic	1			
Triallelic	2	28		
Quadriallelic	3	56		
Pentaallelic	4	170		
Hexaallelic	5	56		
Septemallelic	6	24		
Octoallelic	7	8		

In this table,  is an estimated vector of the genotypic values of different composite diplotypes and the residual variance. The largest log-likelihood and/or the smallest AIC or BIC value calculated is thought to correspond to the most likely risk haplotypes and the optimal number of risk haplotypes.

### Hypothesis Tests

For a given data set, testing the existence of functional haplotypes is a first step. This can be done by formulating the following hypotheses:



*H*_1 _: At least one of equality in *H*_0 _does not hold The log-likelihood ratio (LR) is then calculated by plugging the estimated parameters into the likelihood under the *H*_0 _and *H*_1_, respectively. The LR can be viewed as being asymptotically *χ*^2^-distributed with (*k *+ 1)^2 ^- 1 degrees of freedom.

After a significant haplotype effect is detected, a series of further tests are performed for the significance of additive, dominance and imprinting effects triggered by haplotypes. The null hypotheses under each of these tests can be formulated by setting the effect being tested to be equal to zero. For example, under the triallelic model, the null hypothesis for testing the imprinting effect of the haplotypesis expressed as



In practice, it is also interesting to test each of the additive genetic effects, each of the dominance effects and each of the imprinting effects for the tri-and quadriallelic models. The estimates of the parameters under the null hypotheses can be obtained with the same EM algorithm derived for the alternative hypotheses but with a constraint of the tested effect equal to zero. The log-likelihood ratio test statistics for each hypothesis is thought to asymptotically follow a *χ*^2^-distributed with the degree of freedom equal to the difference of the numbers of the parameters being tested under the null and alternative hypotheses.

## Competing interests

The authors declare that they have no competing interests.

## Authors' contributions

SW, CW, and AB contributed equally to this manuscript. SW, CW, AB, YL derived the algorithm and performed the data analysis. RBF, MRW, RS, LK designed the experiment and collected the data. MMC provided statistical advice and help. RW conceived the model and wrote the paper. AB provided final modifications to the paper. All authors have read and approved the final manuscript.

## Supplementary Material

Additional file 1 **Observed genotypes, underlying diplotypes, and diplotype frequencies under biallelic and ocoallelic models**. Two tables are provided describing genotypes, diplotypes, and diplotype frequencies for 27 genotypes at three SNPs, and genotypic values of composite diplotypes under the biallelic (assuming that 111 is the risk haplotype and the others are the non-risk haplotype) and ocoallelic models. Click here for file

Additional file 2 **EM algorithm details for calculating parameter estimation**. EM Algorithms for estimating haplotype frequencies and for estimating quantitative genetic parameters. Click here for file
