# Supplementary material for: Modeling genetic imprinting effects of DNA sequences with multilocus polymorphism data
Source: Algorithms Mol Biol. 2009 Aug 11;4:11. doi: 10.1186/1748-7188-4-11 (PMC2739217; doi:10.1186/1748-7188-4-11)
Supplement: Additional file 1 — Observed genotypes, underlying diplotypes, and diplotype frequencies under biallelic and ocoallelic models. Two tables are provided describing genotypes, diplotypes, and diplotype frequencies for 27 genotypes at three SNPs, and genotypic values of composite diplotypes under the biallelic (assuming that 111 is the risk haplotype and the others are the non-risk haplotype) and ocoallelic models. [file 1748-7188-4-11-S1.pdf]

Tables: Genotypes, diplotypes, and diplotype frequencies for 27 genotypes at three SNPs, and genotypic values of composite diplotypes under the biallelic (assuming that 111 is the risk haplotype and the others are the non-risk haplotype) and ocoallelic models.

	Diplotype		Composite Diplotype			
			Biallelic Model		Ocoallelic Model	
Genotype	Configuration	Frequency	Symbol	Value	Symbol	Value
11/11/11	111|111	$p^M_{111}p^P_{111}$	R_1R_1	μ_{11}	R_1R_1	μ_{11}
11/11/10	111|110+110|111	$p^M_{111}p^P_{110} + p^M_{110}p^P_{111}$	$R_1R_0 + R_0R_1$	μ_{10}, μ_{01}	$R_1R_2 + R_2R_1$	μ_{12}, μ_{21}
11/11/00	110|110	$p^M_{110}p^P_{110}$	R_0R_0	μ_{00}	R_2R_2	μ_{22}
11/10/11	111|101+101|111	$p^P_{111}p^P_{101} + p^P_{101}p^P_{111}$	$R_1R_0 + R_0R_1$	μ_{10}, μ_{01}	$R_1R_3 + R_3R_1$	μ_{13}, μ_{31}
11/10/10	{ 111|100 + 100|111	{ $p^M_{111}p^P_{100} + p^M_{100}p^P_{111}$	{ $R_1R_0 + R_0R_1$	{ μ_{10}, μ_{01}	{ $R_1R_4 + R_4R_1$	{ μ_{14}, μ_{41}
	110|101 + 101|110	$p^M_{110}p^P_{101} + p^M_{101}p^P_{110}$	R_0R_0	μ_{00}	$R_2R_3 + R_3R_2$	μ_{23}, μ_{32}
11/10/00	110|100+100|1110	$p^M_{110}p^P_{100} + p^M_{100}p^P_{110}$	R_0R_0	μ_{00}	$R_2R_4 + R_4R_2$	μ_{24}, μ_{42}
11/00/11	101|101	$p^M_{101}p^P_{101}$	R_0R_0	μ_{00}	R_3R_3	μ_{33}
11/00/10	101|100+100|101	$p^M_{101}p^P_{100} + p^M_{100}p^P_{101}$	R_0R_0	μ_{00}	$R_3R_4 + R_4R_3$	μ_{34}, μ_{43}
11/00/00	100|100	$p^M_{100}p^P_{100}$	R_0R_0	μ_{00}	R_4R_4	μ_{44}
10/11/11	111|011+011|111	$p^M_{111}p^P_{011} + p^M_{011}p^P_{111}$	$R_1R_0 + R_0R_1$	μ_{10}, μ_{01}	$R_1R_5 + R_5R_1$	μ_{15}, μ_{51}
10/11/10	{ 111|010 + 010|111	{ $p^M_{111}p^P_{010} + p^M_{010}p^P_{111}$	{ $R_1R_0 + R_0R_1$	{ μ_{10}, μ_{01}	{ $R_1R_6 + R_6R_1$	{ μ_{16}, μ_{61}
	110|011 + 011|110	$p^M_{110}p^P_{011} + p^M_{011}p^P_{110}$	R_0R_0	μ_{00}	$R_2R_5 + R_5R_2$	μ_{52}, μ_{25}
10/11/00	110|010+010|110	$p^M_{110}p^P_{010} + p^M_{010}p^P_{110}$	R_0R_0	μ_{00}	$R_2R_6 + R_6R_2$	μ_{26}, μ_{62}
10/10/11	{ 111|001 + 001|111	{ $p^M_{111}p^P_{001} + p^M_{001}p^P_{111}$	{ $R_1R_0 + R_0R_1$	{ μ_{10}, μ_{01}	{ $R_1R_7 + R_7R_1$	{ μ_{17}, μ_{71}
	101|011 + 011|101	$p^M_{101}p^P_{011} + p^M_{011}p^P_{101}$	R_0R_0	μ_{00}	$R_3R_5 + R_5R_3$	μ_{35}, μ_{53}
10/10/10	{ 111|000 + 000|111	{ $p^M_{111}p^P_{000} + p^M_{000}p^P_{111}$	{ $R_1R_0 + R_0R_1$	{ μ_{10}, μ_{01}	{ $R_1R_8 + R_8R_1$	{ μ_{18}, μ_{81}
	110|001 + 001|110	$p^M_{110}p^P_{001} + p^M_{001}p^P_{110}$	R_0R_0	μ_{00}	$R_2R_7 + R_7R_2$	μ_{27}, μ_{72}
	101|010 + 010|101	$p^M_{101}p^P_{010} + p^M_{010}p^P_{101}$	R_0R_0	μ_{00}	$R_3R_6 + R_6R_3$	μ_{36}, μ_{63}
	100|001 + 001|100	$p^M_{100}p^P_{011} + p^M_{011}p^P_{100}$	R_0R_0	μ_{00}	$R_4R_5 + R_5R_4$	μ_{45}, μ_{54}

Genotype	Diplotype: Configuration	Diplotype: Frequency	Composite Diplotype, Biallelic Model: Symbol	Composite Diplotype, Biallelic Model: Value	Composite Diplotype, Ocoallelic Model: Symbol	Composite Diplotype, Ocoallelic Model: Value
10/10/00	110|000 + 000|110	$p^M_{110}p^P_{000} + p^M_{000}p^P_{110}$	R_0R_0	μ_{00}	$R_2R_8 + R_8R_2$	μ_{28}, μ_{82}
	100|010 + 010|100	$p^M_{100}p^P_{010} + p^M_{010}p^P_{100}$	R_0R_0	μ_{00}	$R_4R_6 + R_6R_4$	μ_{46}, μ_{64}
10/00/11	101|001 + 001|101	$p^M_{101}p^P_{001} + p^M_{001}p^P_{101}$	R_0R_0	μ_{00}	$R_3R_7 + R_7R_3$	μ_{37}, μ_{73}
10/00/10	101|000 + 000|101	$p^M_{101}p^P_{000} + p^M_{00}p^P_{101}$	R_0R_0	μ_{00}	$R_3R_8 + R_8R_3$	μ_{38}, μ_{83}
	100|001 + 001|100	$p^M_{100}p^P_{001} + p^M_{001}p^P_{100}$	R_0R_0	μ_{00}	$R_4R_7 + R_7R_4$	μ_{47}, μ_{74}
10/00/00	100|000 + 000|100	$p^M_{100}p^P_{000} + p^M_{000}p^P_{100}$	R_0R_0	μ_{00}	$R_4R_8 + R_8R_4$	μ_{48}, μ_{84}
00/11/11	011|011	$p^M_{011}p^P_{011}$	R_0R_0	μ_{00}	R_5R_5	μ_{55}
00/11/10	011|010 + 010|011	$p^M_{011}p^P_{010} + p^M_{010}p^P_{011}$	R_0R_0	μ_{00}	$R_5R_6 + R_6R_5$	μ_{56}, μ_{65}
00/11/00	010|010	$p^M_{010}p^P_{010}$	R_0R_0	μ_{00}	R_6R_6	μ_{66}
00/10/11	011|001 + 001|011	$p^M_{011}p^P_{001} + p^M_{001}p^P_{011}$	R_0R_0	μ_{00}	$R_5R_7 + R_7R_5$	μ_{57}, μ_{75}
00/10/10	011|000 + 000|011	$p^M_{011}p^P_{000} + p^M_{000}p^P_{011}$	R_0R_0	μ_{00}	$R_5R_8 + R_8R_5$	μ_{58}, μ_{85}
	010|001 + 001|010	$p^M_{010}p^P_{001} + p^M_{001}p^P_{010}$	R_0R_0	μ_{00}	$R_6R_7 + R_7R_6$	μ_{67}, μ_{76}
00/10/00	010|000	$p^M_{010}p^P_{000} + p^M_{000}p^P_{010}$	R_0R_0	μ_{00}	$R_6R_8 + R_8R_6$	μ_{68}, μ_{86}
00/00/11	001|001	$p^M_{001}p^P_{001}$	R_0R_0	μ_{00}	R_7R_7	μ_{77}
00/00/10	001|000	$p^M_{001}p^P_{000} + p^M_{000}p^P_{001}$	R_0R_0	μ_{00}	$R_7R_8 + R_8R_7$	μ_{78}, μ_{87}
00/00/00	000|000	$p^M_{000}p^P_{000}$	R_0R_0	μ_{00}	R_8R_8	μ_{88}
