# Supplementary material for: Modeling genetic imprinting effects of DNA sequences with multilocus polymorphism data
Source: Algorithms Mol Biol. 2009 Aug 11;4:11. doi: 10.1186/1748-7188-4-11 (PMC2739217; doi:10.1186/1748-7188-4-11)
Supplement: Additional file 2 — EM algorithm details for calculating parameter estimation. EM Algorithms for estimating haplotype frequencies and for estimating quantitative genetic parameters. [file 1748-7188-4-11-S2.pdf]

The EM Algorithms

Estimating Haplotype Frequencies

Based on likelihood (8), the EM algorithm is derived to estimate haplotype frequencies. In the E step, calculate the proportions of a particular diplotype within double or triple heterozygous genotypes in the population of sex s by

$$\phi_1^s = \frac{p_{111}^s p_{100}^s}{p_{111}^s p_{100}^s + p_{101}^s p_{110}^s}, \bar{\phi}_1^s = 1 - \phi_1^s \qquad \text{for genotype } 11/10/10$$

$$\phi_2^s = \frac{p_{111}^s p_{010}^s}{p_{111}^s p_{010}^s + p_{011}^s p_{110}^s}, \bar{\phi}_2^s = 1 - \phi_2^s \qquad \text{for genotype } 10/11/10$$

$$\phi_3^s = \frac{p_{111}^s p_{001}^s}{p_{111}^s p_{001}^s + p_{101}^s p_{011}^s}, \bar{\phi}_3^s = 1 - \phi_3^s \qquad \text{for genotype } 10/10/11$$

$$\begin{cases} \phi_4^s &= \dfrac{p_{111}^s p_{000}^s}{p_{111}^s p_{000}^s + p_{101}^s p_{010}^s + p_{110}^s p_{001}^s + p_{100}^s p_{011}^s} \\ \bar{\phi}_4^s &= \dfrac{p_{101}^s p_{010}^s}{p_{111}^s p_{000}^s + p_{101}^s p_{010}^s + p_{110}^s p_{001}^s + p_{100}^s p_{011}^s} \\ \bar{\bar{\phi}}_4^s &= \dfrac{p_{110}^s p_{001}^s}{p_{111}^s p_{000}^s + p_{101}^s p_{010}^s + p_{110}^s p_{001}^s + p_{100}^s p_{011}^s} \end{cases} \qquad \text{for genotype } 10/10/10$$

$$\phi_5^s = \frac{p_{110}^s p_{000}^s}{p_{110}^s p_{000}^s + p_{100}^s p_{010}^s}, \bar{\phi}_5^s = 1 - \phi_5^s \qquad \text{for genotype } 10/10/00$$

$$\phi_6^s = \frac{p_{101}^s p_{000}^s}{p_{101}^s p_{000}^s + p_{001}^s p_{100}^s}, \bar{\phi}_6^s = 1 - \phi_6^s \qquad \text{for genotype } 10/00/10$$

$$\phi_7^s = \frac{p_{011}^s p_{000}^s}{p_{011}^s p_{000}^s + p_{001}^s p_{010}^s}, \bar{\phi}_7^s = 1 - \phi_7^s \qquad \text{for genotype } 00/10/10$$

In the M step, estimate the haplotype frequencies with the calculated relative proportions

by

$$
\begin{aligned}
\hat{p}^s_{111} &= \tfrac{1}{2n}(2n^s_{11/11/11} + n^s_{11/11/10} + n^s_{11/10/11} + n^s_{10/11/11} \\
&\qquad + \phi^s_1 n^s_{11/10/10} + \phi^s_2 n^s_{10/11/10} + \phi^s_3 n^s_{10/10/11} + \phi^s_4 n^s_{10/10/10}) \\
\hat{p}^s_{110} &= \tfrac{1}{2n}(2n^s_{11/11/00} + n^s_{11/11/10} + n^s_{11/10/00} + n^s_{10/11/00} \\
&\qquad + \bar{\phi}_1 n^s_{11/10/10} + \bar{\phi}_2 n^s_{10/11/10} + \bar{\bar{\phi}}^s_4 n^s_{10/10/10} + \phi^s_5 n^s_{10/10/00}) \\
\hat{p}^s_{101} &= \tfrac{1}{2n}(2n^s_{11/00/11} + n^s_{11/10/11} + n^s_{11/00/10} + n^s_{10/00/11} \\
&\qquad + \bar{\phi}_1 n^s_{11/10/10} + \bar{\phi}_3 n^s_{10/10/11} + \bar{\phi}_4 n^s_{10/10/10} + \phi^s_6 n^s_{10/00/10}) \\
\hat{p}^s_{100} &= \tfrac{1}{2n}[2n^s_{11/00/00} + n^s_{11/10/00} + n^s_{11/00/10} + n^s_{10/00/00} \\
&\qquad + \phi^s_1 n^s_{11/10/10} + (1 - \phi^s_4 - \bar{\phi}^s_4 - \bar{\bar{\phi}}^s_4) n^s_{10/10/10} + \bar{\phi}_5 n^s_{10/10/00} + \bar{\phi}_6 n^s_{10/00/10}] \\
\hat{p}^s_{011} &= \tfrac{1}{2n}[2n^s_{00/11/11} + n^s_{10/11/11} + n^s_{00/10/11} + n^s_{00/11/10} \\
&\qquad + \bar{\phi}_2 n^s_{10/11/10} + \bar{\phi}_3 n^s_{10/10/11} + (1 - \phi^s_4 - \bar{\phi}^s_4 - \bar{\bar{\phi}}^s_4) n^s_{10/10/10} + \phi^s_7 n^s_{00/10/10}] \\
\hat{p}^s_{010} &= \tfrac{1}{2n}(2n^s_{00/11/00} + n^s_{10/11/00} + n^s_{00/11/10} + n^s_{00/10/00} \\
&\qquad + \phi^s_2 n^s_{10/11/10} + \bar{\phi}_4 n^s_{10/10/10} + \bar{\phi}_5 n^s_{10/10/00} + \bar{\phi}_7 n^s_{00/10/10}) \\
\hat{p}^s_{001} &= \tfrac{1}{2n}(2n^s_{00/00/11} + n^s_{10/00/11} + n^s_{00/10/11} + n^s_{00/00/10} \\
&\qquad + \phi^s_3 n^s_{10/10/11} + \bar{\bar{\phi}}_4 n^s_{10/10/10} + \bar{\phi}_6 n^s_{10/00/10} + \bar{\phi}_7 n^s_{00/10/10}) \\
\hat{p}^s_{000} &= \tfrac{1}{2n}(2n^s_{00/00/00} + n^s_{00/00/10} + n^s_{00/10/00} + n^s_{10/00/00} \\
&\qquad + \phi^s_5 n^s_{00/10/10} + \phi^s_6 n^s_{10/00/10} + \phi^s_7 n^s_{10/10/00} + \phi^s_4 n^s_{10/10/10}).
\end{aligned}
$$

The E and M steps are iterated until the estimates of haplotype frequencies are stable.

Estimating Quantitative Genetic Parameters

Based on likelihood (9), the EM algorithm is derived to estimate the genetic values of composite diplotypes and residual variance. In the E step, calculate the posterior probabilities with which a double or triple heterozygous subject i is a particular diplotype, expressed

as

$$\Psi_{1i}^{10} = \frac{\psi_1 f_{10}(y_i)}{\psi_1 f_{10}(y_i) + \bar{\psi}_1 f_{01}(y_i)},\ \Psi_{1i}^{01} = 1 - \Psi_{1i}^{10} \qquad \text{for genotype 11/11/10}$$

$$\Psi^{10}2i = \frac{\psi_2 f_{10}(y_i)}{\psi_2 f_{10}(y_i) + \bar{\psi}_2 f_{01}(y_i)},\ \Psi_{2i}^{01} = 1 - \Psi_{2i}^{10} \qquad \text{for genotype 11/10/11}$$

$$\left\{\begin{array}{lcl} \Psi_{3i}^{10} & = & \dfrac{\psi_3 f_{10}(y_i)}{\psi_3 f_{10}(y_i) + \bar{\psi}_3 f_{01}(y_i) + \bar{\bar{\psi}}_3 f_{00}(y_i)}, \\ \Psi_{3i}^{01} & = & \dfrac{\bar{\psi}_3 f_{01}(y_i)}{\psi_3 f_{10}(y_i) + \bar{\psi}_3 f_{01}(y_i) + \bar{\bar{\psi}}_3 f_{00}(y_i)}, \\ \Psi_{3i}^{00} & = & \dfrac{\bar{\bar{\psi}}_3 f_{00}(y_i)}{\psi_3 f_{10}(y_i) + \bar{\psi}_3 f_{01}(y_i) + \bar{\bar{\psi}}_3 f_{00}(y_i)}, \end{array}\right. \qquad \text{for genotype 11/10/10}$$

$$\Psi_{4i}^{10} = \frac{\psi_4 f_{10}(y_i)}{\psi_4 f_{10}(y_i) + \bar{\psi}_4 f_{01}(y_i)},\ \Psi_{4i}^{01} = 1 - \Psi_{4i}^{10} \qquad \text{for genotype 10/11/11}$$

$$\left\{\begin{array}{lcl} \Psi_{5i}^{10} & = & \dfrac{\psi_5 f_{10}(y_i)}{\psi_5 f_{10}(y_i) + \bar{\psi}_5 f_{01}(y_i) + \bar{\bar{\psi}}_5 f_{00}(y_i)}, \\ \Psi_{5i}^{01} & = & \dfrac{\bar{\psi}_5 f_{01}(y_i)}{\psi_5 f_{10}(y_i) + \bar{\psi}_5 f_{01}(y_i) + \bar{\bar{\psi}}_5 f_{00}(y_i)}, \\ \Psi_{5i}^{00} & = & \dfrac{\bar{\bar{\psi}}_5 f_{00}(y_i)}{\psi_5 f_{10}(y_i) + \bar{\psi}_5 f_{01}(y_i) + \bar{\bar{\psi}}_5 f_{00}(y_i)}, \end{array}\right. \qquad \text{for genotype 10/11/10}$$

$$\left\{\begin{array}{lcl} \Psi_{6i}^{10} & = & \dfrac{\psi_6 f_{10}(y_i)}{\psi_6 f_{10}(y_i) + \bar{\psi}_6 f_{01}(y_i) + \bar{\bar{\psi}}_6 f_{00}(y_i)}, \\ \Psi_{6i}^{01} & = & \dfrac{\bar{\psi}_6 f_{01}(y_i)}{\psi_6 f_{10}(y_i) + \bar{\psi}_6 f_{01}(y_i) + \bar{\bar{\psi}}_6 f_{00}(y_i)}, \\ \Psi_{6i}^{00} & = & \dfrac{\bar{\bar{\psi}}_6 f_{00}(y_i)}{\psi_6 f_{10}(y_i) + \bar{\psi}_6 f_{01}(y_i) + \bar{\bar{\psi}}_6 f_{00}(y_i)}, \end{array}\right. \qquad \text{for genotype 10/10/11}$$

$$\left\{\begin{array}{lcl} \Psi_{7i}^{10} & = & \dfrac{\psi_7 f_{10}(y_i)}{\psi_7 f_{10}(y_i) + \bar{\psi}_7 f_{01}(y_i) + \bar{\bar{\psi}}_7 f_{00}(y_i)}, \\ \Psi_{7i}^{01} & = & \dfrac{\bar{\psi}_7 f_{01}(y_i)}{\psi_7 f_{10}(y_i) + \bar{\psi}_7 f_{01}(y_i) + \bar{\bar{\psi}}_7 f_{00}(y_i)}, \\ \Psi_{7i}^{00} & = & \dfrac{\bar{\bar{\psi}}_7 f_{00}(y_i)}{\psi_7 f_{10}(y_i) + \bar{\psi}_7 f_{01}(y_i) + \bar{\bar{\psi}}_7 f_{00}(y_i)}, \end{array}\right. \qquad \text{for genotype 10/10/10}$$

In the M step, estimate the quantitative genetic parameters, Ω_q, with the calculated

posterior probabilities by

$$\mu_{11} = \frac{\sum_{i=1}^{n_{11/11/11}} y_i}{n_{11/11/11}},$$

$$\mu_{10} = \frac{\sum_{i=1}^{n_{11/11/10}} \Psi_{1i}^{10} y_i + \sum_{i=1}^{n_{11/10/11}} \Psi_{2i}^{10} y_i + \sum_{i=1}^{n_{11/10/10}} \Psi_{3i}^{10} y_i + \sum_{i=1}^{n_{10/11/11}} \Psi_{4i}^{10} y_i + \sum_{i=1}^{n_{10/11/10}} \Psi_{5i}^{10} y_i + \sum_{i=1}^{n_{10/10/11}} \Psi_{6i}^{10} y_i + \sum_{i=1}^{n_{10/10/10}} \Psi_{7i}^{10} y_i}{\sum_{i=1}^{n_{11/11/10}} \Psi_{1i}^{10} + \sum_{i=1}^{n_{11/10/11}} \Psi_{2i}^{10} + \sum_{i=1}^{n_{11/10/10}} \Psi_{3i}^{10} + \sum_{i=1}^{n_{10/11/11}} \Psi_{4i}^{10} + \sum_{i=1}^{n_{10/11/10}} \Psi_{5i}^{10} + \sum_{i=1}^{n_{10/10/11}} \Psi_{7i}^{10} + \sum_{i=1}^{n_{10/10/10}} \Psi_{7i}^{10}}$$

$$\mu_{01} = \frac{\sum_{i=1}^{n_{11/11/10}} \Psi_{1i}^{01} y_i + \sum_{i=1}^{n_{11/10/11}} \Psi_{2i}^{01} y_i + \sum_{i=1}^{n_{11/10/10}} \Psi_{3i}^{01} y_i + \sum_{i=1}^{n_{10/11/11}} \Psi_{4i}^{01} y_i + \sum_{i=1}^{n_{10/11/10}} \Psi_{5i}^{01} y_i + \sum_{i=1}^{n_{10/10/11}} \Psi_{6i}^{01} y_i + \sum_{i=1}^{n_{10/10/10}} \Psi_{7i}^{01} y_i}{\sum_{i=1}^{n_{11/11/10}} \Psi_{1i}^{01} + \sum_{i=1}^{n_{11/10/11}} \Psi_{2i}^{01} + \sum_{i=1}^{n_{11/10/10}} \Psi_{3i}^{01} + \sum_{i=1}^{n_{10/11/11}} \Psi_{4i}^{01} + \sum_{i=1}^{n_{10/11/10}} \Psi_{5i}^{01} + \sum_{i=1}^{n_{10/10/11}} \Psi_{7i}^{01} + \sum_{i=1}^{n_{10/10/10}} \Psi_{7i}^{01}}$$

$$\mu_{00} = \frac{\sum_{i=1}^{m} y_i + \sum_{i=1}^{n_{11/10/10}} \Psi_{3i}^{00} y_i + \sum_{i=1}^{n_{10/11/10}} \Psi_{5i}^{00} y_i + \sum_{i=1}^{n_{10/10/11}} \Psi_{6i}^{00} y_i + \sum_{i=1}^{n_{10/10/10}} \Psi_{7i}^{00} y_i}{m + + \sum_{i=1}^{n_{11/10/10}} \Psi_{3i}^{00} + \sum_{i=1}^{n_{10/11/10}} \Psi_{5i}^{00} + \sum_{i=1}^{n_{10/10/11}} \Psi_{6i}^{00} + \sum_{i=1}^{n_{10/10/10}} \Psi_{7i}^{00}},$$

$$\begin{aligned}
\sigma^2 = \frac{1}{n}[& \sum_{i=1}^{n_{11/11/11}} (y_i - \mu_{11})^2 + \sum_{i=1}^{n_{11/11/10}} (y_i - \mu_{10})^2 \Psi_{1i}^{10} + \sum_{i=1}^{n_{11/10/11}} (y_i - \mu_{10})^2 \Psi_{2i}^{10} + \sum_{i=1}^{n_{11/10/10}} (y_i - \mu_{10})^2 \Psi_{3i}^{10} \\
+ & \sum_{i=1}^{n_{10/11/11}} (y_i - \mu_{10})^2 \Psi_{4i}^{10} + \sum_{i=1}^{n_{10/11/10}} (y_i - \mu_{10})^2 \Psi_{5i}^{10} + \sum_{i=1}^{n_{10/10/11}} (y_i - \mu_{10})^2 \Psi_{7i}^{10} + \sum_{i=1}^{n_{10/10/10}} (y_i - \mu_{10})^2 \Psi_{7i}^{10} \\
+ & \sum_{i=1}^{n_{11/11/10}} (y_i - \mu_{01})^2 \Psi_{1i}^{01} + \sum_{i=1}^{n_{11/10/11}} (y_i - \mu_{01})^2 \Psi_{2i}^{01} + \sum_{i=1}^{n_{11/10/10}} (y_i - \mu_{01})^2 \Psi_{3i}^{01} + \sum_{i=1}^{n_{10/11/11}} (y_i - \mu_{01})^2 \Psi_{4i}^{01} \\
+ & \sum_{i=1}^{n_{10/11/10}} (y_i - \mu_{01})^2 \Psi_{5i}^{01} + \sum_{i=1}^{n_{10/10/11}} (y_i - \mu_{01})^2 \Psi_{7i}^{01} + \sum_{i=1}^{n_{10/10/10}} (y_i - \mu_{01})^2 \Psi_{7i}^{01} + \sum_{i=1}^{m} (y_i - \mu_{00})^2 \\
+ & \sum_{i=1}^{n_{11/10/10}} (y_i - \mu_{00})^2 \Psi_{3i}^{00} + \sum_{i=1}^{n_{10/11/10}} (y_i - \mu_{00})^2 \Psi_{5i}^{00} + \sum_{i=1}^{n_{10/10/11}} (y_i - \mu_{00})^2 \Psi_{6i}^{00} + \sum_{i=1}^{n_{10/10/10}} (y_i - \mu_{00})^2 \Psi_{7i}^{00}.
\end{aligned}$$

The E and M steps are iterated until the estimates of haplotype frequencies are stable.
